# The CRISPR/Cas9 system inactivates latent HIV-1 proviral DNA

**DOI:** 10.1186/s12977-015-0150-z

**Published:** 2015-02-27

**Authors:** Weijun Zhu, Rongyue Lei, Yann Le Duff, Jian Li, Fei Guo, Mark A Wainberg, Chen Liang

**Affiliations:** MOH Key Laboratory of Systems Biology of Pathogens and AIDS Research Center, Institute of Pathogen Biology, Chinese Academy of Medical Sciences & Peking Union Medical College, Beijing, 100730 PR China; McGill University AIDS Centre, Lady Davis Institute, Jewish General Hospital, Montreal, H3T 1E2 Canada; Departments of Medicine, Microbiology & Immunology, McGill University, Montreal, H3A 2B4 Canada

**Keywords:** HIV-1, Provirus, Reservoir, CRISPR/Cas9, Genome editing

## Abstract

**Background:**

Highly active antiretroviral therapy (HAART) has transformed HIV-1 infection from a deadly disease to a manageable chronic illness, albeit does not provide a cure. The recently developed genome editing system called CRISPR/Cas9 offers a new tool to inactivate the integrated latent HIV-1 DNA and may serve as a new avenue toward cure.

**Findings:**

We tested 10 sites in HIV-1 DNA that can be targeted by CRISPR/Cas9. The engineered CRISPR/Cas9 system was introduced into the JLat10.6 cells that are latently infected by HIV-1. The sequencing results showed that each target site in HIV-1 DNA was efficiently mutated by CRISPR/Cas9 with the target site in the second exon of Rev (called T10) exhibiting the highest degree of mutation. As a result, HIV-1 gene expression and virus production were significantly diminished with T10 causing a 20-fold reduction.

**Conclusions:**

The CRISPR/Cas9 complex efficiently mutates and deactivates HIV-1 proviral DNA in latently infected Jurkat cells. Our results also revealed a highly efficient Cas9 target site within the second exon of Rev that represents a promising target to be further explored in the CRISPR/Cas9-based cure strategy.

## Findings

Current antiretroviral therapy (ART) enables control of HIV infection at both the individual level and on a global scale. As a result, steady declines have been seen in both numbers of new HIV infections as well as in HIV mortality rates [1,2]. Unfortunately, a cure of HIV infection has not yet been attained. One block to this ultimate goal is the persistence of HIV reservoirs that cannot be cleared by current ART [3]. The establishment of viral reservoirs requires the integration of HIV DNA into cellular genome [4]. Theoretically, deleting or deactivating proviral DNA should eliminate the source of HIV persistence, and may thus provide a valuable tool toward cure.

Two approaches have been explored in this context. One is based on the zinc-finger nucleases (ZFNs) that were engineered to recognize and cleave specific HIV DNA sequences [5]. The second approach is called TALENS (transcription activator-like effector nucleases) that program transcription factors to recognize specific DNA sequences [6]. Both approaches exploit the principle of protein-DNA recognition that often involves a certain degree of ambiguity. A recent development in genome editing is the CRISPR (Type II microbial clustered regularly interspaced short palindromic repeat)/Cas9 system that utilizes a guide RNA strand to recognize and mutate target DNA; this therefore now offers a new strategy to inactivate HIV DNA [7-10].

This CRISPR/Cas9 system arms a DNA endonuclease called Cas9 with an RNA component that utilizes a 20-nucleotide guide sequence (gRNA) to recognize a specific DNA site [7,8]. Cleavage of DNA by Cas9 generates double-stranded DNA breaks that, upon non-homologous end joining (NHEJ) repair, cause insertions or deletions at the DNA target sites. This approach has been demonstrated highly specific and efficient in targeting multiple cellular genes and has been further developed for genome-wide screens to study gene function [11-14]. In this study, we have tested the efficiency of the CRISPR/Cas9 system in inactivating the integrated HIV-1 DNA in a HIV-GFP Jurkat cell line called JLat10.6 that was developed to study HIV latency [15].

**Figure 1:**
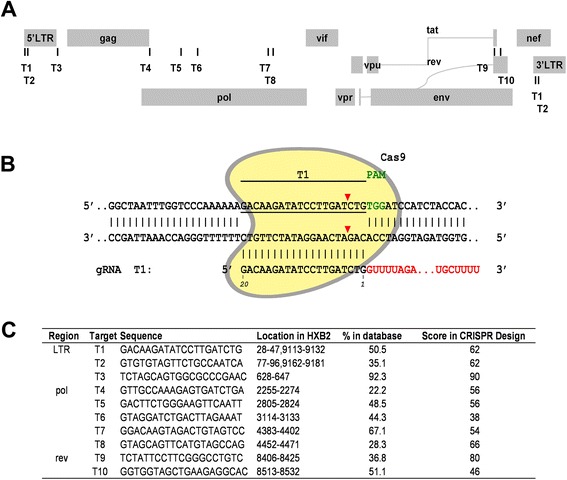
**Illustration of the ten HIV-1 guide RNAs tested in this study. (A)** Locations of the 10 guide RNAs (T1 to T10) in HIV-1 genome. **(B)** Schematic depiction of binding of the T1 gRNA guide sequence (20 nucleotides, underlined) to the HIV-1 DNA in the context of Cas9 (in yellow). The PAM (protospacer adjacent motif) is highlighted in green letters. The red arrow indicates the cleavage site by Cas9. **(C)** Sequences of the 10 target sites in the genome HIV-1 strain HXB2 (T1 to T10). Conservation of each target sequence in HIV database is presented. The CRISPR score of each gRNA was calculated using the program at http://www.genome-engineering.org.

**Figure 2:**
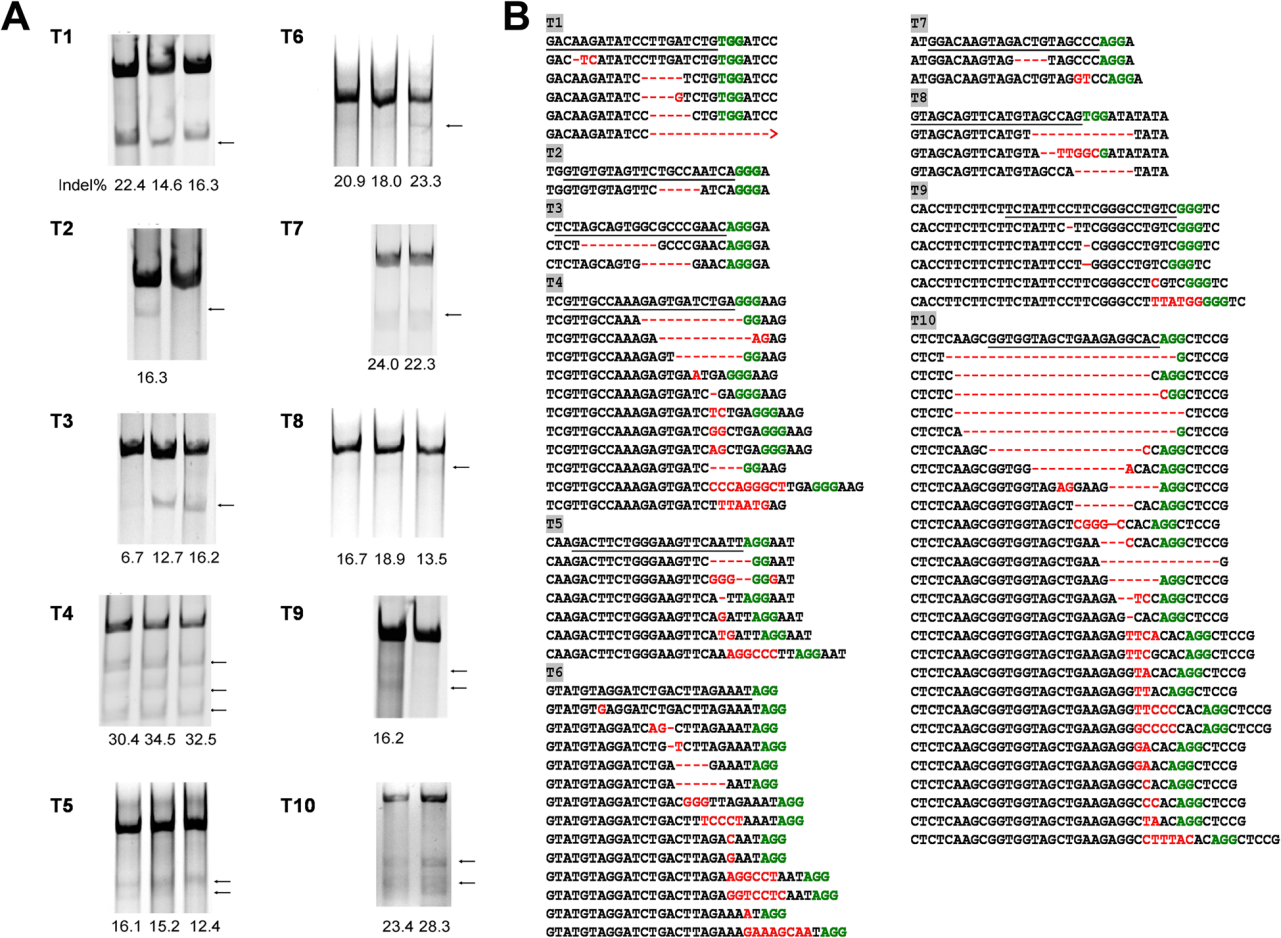
**Analysis of mutations in HIV-1 DNA caused by CRISPR/Cas9 treatment. (A)** Results of the SURVEYOR assays to measure the NHEJ events resulting from HIV-1 guide RNA treatment. Data of two or three independent transfection experiments are shown. The percentage of the mutated DNA (indicated by arrows) is presented at the bottom of each lane. **(B)** Mutations at each gRNA target site. The mutated sequences (deletions, insertions and substitutions) are shown in red letters. The PAMs are highlighted in green letters. The wild type HIV-1 DNA is underlined and shown at the top of each aligned sequence panel.

This JLat10.6 HIV-latent cell line harbors the full-length viral DNA that is transcription silent in the absence of external stimulation. Treatment with cytokines such as TNF-α activates viral gene expression, which can be measured by monitoring the expression of GFP that has been inserted into HIV-1 DNA as a reporter gene. In order to target HIV-1 DNA, we surveyed the HIV-1 genome and identified more than 80 PAM (protospacer adjacent motif) sites that potentially allow the CRISPR/Cas9 complex to recognize HIV-1 DNA. After considering the conservation of these target DNA sequences across different HIV-1 strains as well as excluding off-target possibilities by blasting the human genome, 10 gRNAs (named T1 to T10) were selected and inserted into the gRNA-cloning vector [8]. Of these 10 target sites, 3 are located within the LTR, 5 in the *pol* gene, and 2 in the second exon of *tat/rev* (Figure 1). We then nucleotransfected these gRNA constructs, together with the hCas9 plasmid DNA (expressing the humanized Cas9 enzyme) [8], into JLat10.6 cells. SURVEYOR assay was first performed to measure the frequency of NHEJ as a result of the targeted Cas9 cleavage of HIV-1 DNA. NHEJ was demonstrated for all 10 gRNAs. The frequency ranged from 10% to 30% (Figure 2A). The targeted viral DNA regions were also amplified by PCR and further examined by sequencing. Both insertions and deletions of various lengths of nucleotides were observed, with the gRNA T10 causing the most types of deletions and insertions (Figure 2B). These data illustrate the effectiveness of the 10 gRNAs in targeting Cas9 to cleave and mutate HIV-1 DNA at distinct regions.

**Figure 3:**
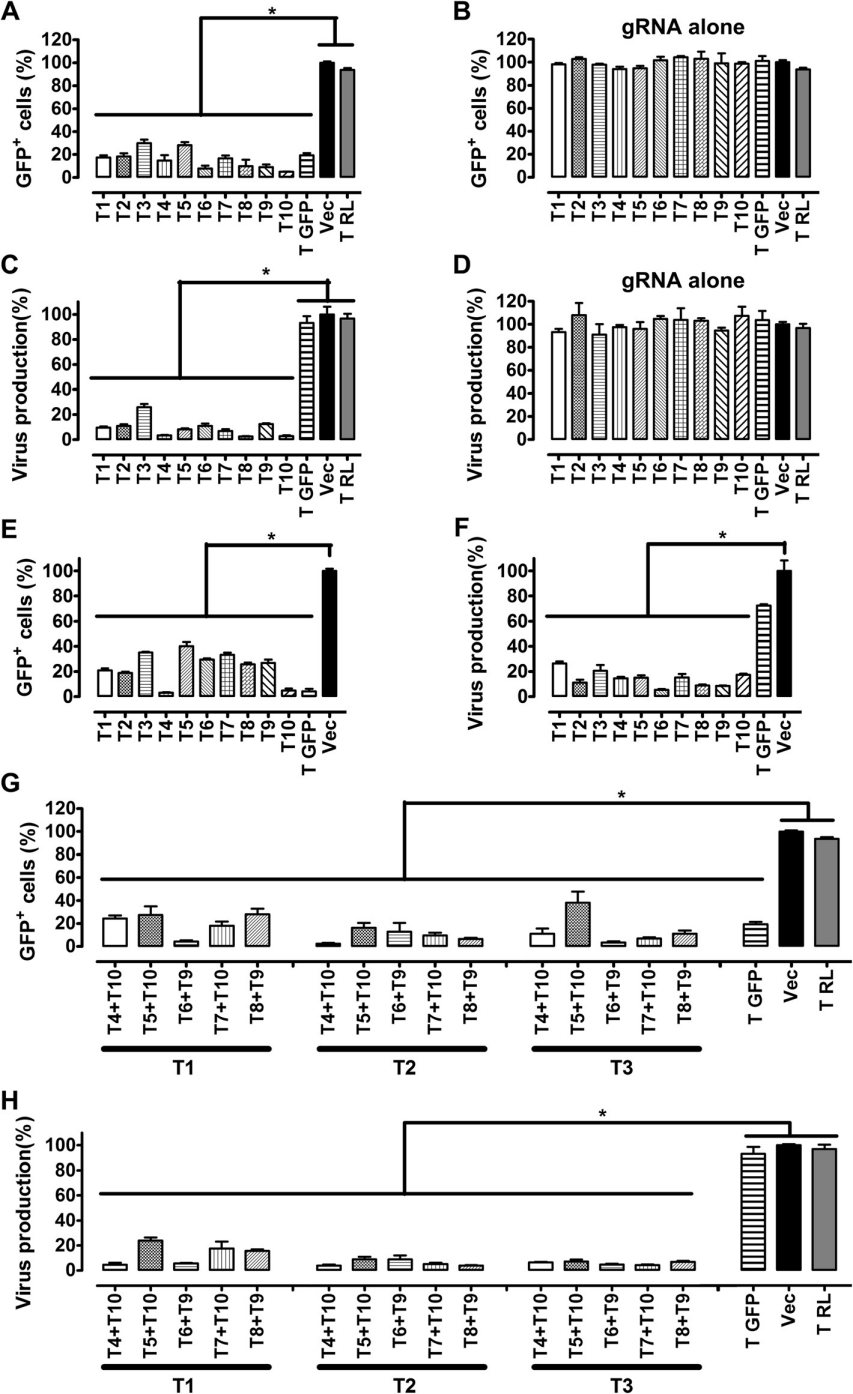
**Suppression of HIV-1 gene expression and virus production by gRNA/Cas9. (A, B)** Effects of gRNA/Cas9 on GFP expression. JLat10.6 cells were transfected with gRNA and hCas9 plasmid DNA using Neon (Life Technologies). TNF-α (10 ng/ml) was added 16 hours after to stimulate HIV-1 gene expression from HIV-1 LTR promoter, which was monitored by flow cytometry. A gRNA targeting the GFP DNA (called T GFP (GTGAACCGCATCGAGCTGAA)) was included as a positive control. The gRNA targeting renilla luciferase DNA (called T RL (GTAGCGCGGTGTATTATACC)) was utilized as a negative control. Results obtained with the empty gRNA vector were arbitrarily set as 100%. The results shown are the average of three independent transfection experiments. Experiments were also performed with gRNA alone (without Cas9) and the results are shown in **(B)**. **(C, D)** Effects of gRNA/Cas9 on virus production. Levels of viruses in the supernatants were determined by ELISA that measures HIV-1 p24 antigen. Results of three independent transfections are shown. **(D)** represents the results of experiments performed with gRNA alone (without Cas9). **(E, F)** Pretreatment with TNF-α does not increase the inhibition of HIV-1 by gRNA/Cas9. JLat10.6 cells were treated with TNF-α (10 ng/ml) for 16 hours before nucleotransfection with gRNA and hCas9 plasmid DNA. GFP-positive cells were scored by flow cytometry and the results are shown in **(E)**. Levels of viruses in the supernatants were determined by HIV-1 p24 ELISA and the results shown in **(F)**. Results are the average of three independent transfections. **(G, H)** Suppression of HIV-1 by multiple gRNAs that target different HIV-1 DNA sites. Three different gRNAs were co-transfected with hCas9 plasmid DNA. HIV-1 gene expression (shown in **(G)**) and virus production (shown in **(H)**) after TNF-α treatment were measured as described above. Results shown are the average of three independent transfection experiments. Asterisks indicate *P* < 0.05 (Mann–Whitney *U* test, SPSS 16.0).

In order to measure the effects of gRNA/Cas9-induced DNA mutations on the function of integrated HIV-1 DNA, we first transfected the JLat10.6 cells with gRNA/Cas9 DNA followed by TNF-α (10 ng/ml) treatment to induce viral gene expression which is monitored by scoring GFP-positive cells by flow cytometry. The results showed that the gRNA targeting GFP DNA (named T GFP) reduced GFP expression by 5-fold (Figure 3A). No effect was observed for a gRNA that targets the renilla luciferase (T RL) DNA. The gRNAs targeting HIV-1 DNA diminished the number of GFP-positive cells to different degrees, ranging from 3-fold (gRNA T3) to 20-fold (gRNA T10) (Figure 3A). These gRNAs alone, without the help of Cas9, exerted no effect on GFP expression (Figure 3B). When levels of HIV-1 in culture supernatants were measured by p24 ELISA, gRNA T3 led to 3-fold diminution as compared to 20-fold decrease associated with gRNAs T4, T8 and T10 (Figure 3C). The gRNAs alone, in the absence of Cas9, did not affect HIV-1 production (Figure 3D), which further confirms that the gRNA molecules act on HIV-1 DNA through arming the Cas9 enzyme. Together, these data demonstrate the high efficacy of the CRISPR/Cas9 system in targeting and inactivating HIV-1 proviral DNA.

In JLat10.6 cells, without any external stimulation, HIV-1 DNA is transcription silent as a result of the inaccessibility of cellular transcriptional machinery to HIV-1 LTR promoter [15,16]. We suspect that this inaccessibility, likely a result of modified chromatin structure, may hinder the ability of the CRISPR/Cas9 complex to target HIV-1 DNA. Were this the case, then pretreatment with HDAC inhibitors such as SAHA may deem necessary to activate HIV-1 gene expression in order to enhance the effectiveness of CRISPR/Cas9 system. We therefore treated the JLat10.6 cells with TNF-α for 16 hours prior to nucleotransfection with the gRNA and hCas9 plasmid DNA. In contrary to our expectation, activation of HIV-1 transcription by TNF-α did not render HIV-1 DNA more susceptible to inhibition by the CRISPR/Cas9 machinery (Figure 3E and F). This observation suggests that the CRISPR/Cas9 system has the ability to access transcription silent HIV-1 DNA without the need to activate viral gene expression by TNF-α or related agents such as SAHA. This property of the CRISPR/Cas9 system supports its possible utility in eradication of integrated HIV-1 DNA from latently infected immune cells such as CD4+ T cells and macrophages, in which HIV-1 transcription is also inert. Given that these HIV-1 latently-infected cells are often non-cycling, further studies are warranted to assess whether cell cycling may affect the efficacy of CRISPR/Cas9.

We next tested whether combinations of different gRNAs further increased the potency of inhibition. This combination strategy is also expected to diminish the chances of HIV-1 to escape from gRNA targeting. Indeed, certain combinations, such as T1/T6/T9, T2/T4/T10 and T3/T6/T9, reduced the numbers of GFP-positive cells and the yields of viruses by more than 24-fold (Figure 3G and H). It is noted that all three combinations contain gRNAs that target *tat/rev*, which indicates that *tat* and *rev* sequences may represent vulnerable sites for CRISPR/Cas9 targeting.

**Figure 4:**
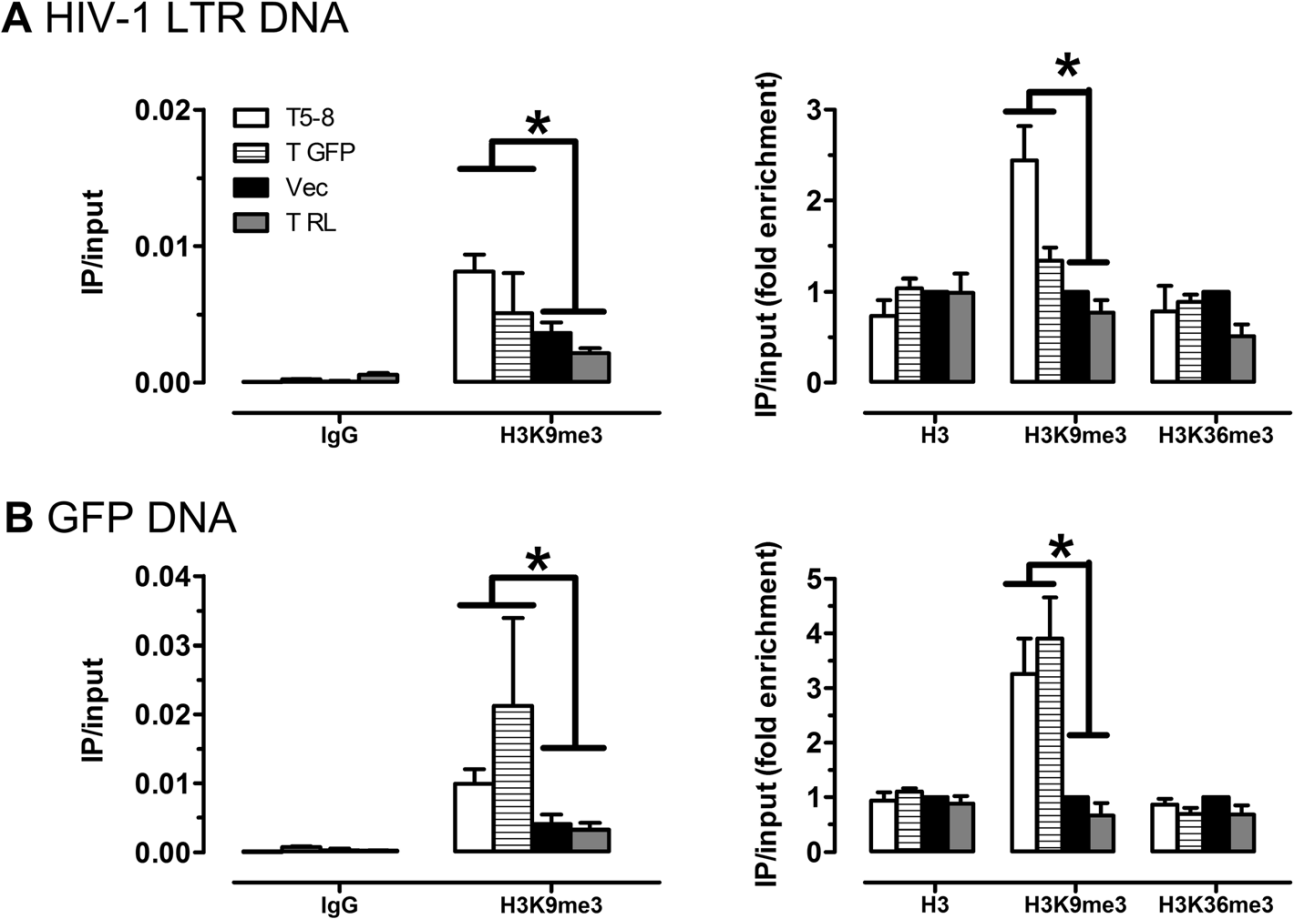
**Effect of gRNA/Cas9 treatment on histone modification at HIV-1 LTR.** JLat10.6 cells were treated with gRNAs that target HIV-1 *pol* gene (T5 to T8). Cells were then harvested and subject to ChIP analysis using control IgG, anti-H3, anti-H3K9me3, or anti-H3K36me3 antibodies. Levels of HIV-1 LTR DNA (shown in **(A)**) or GFP DNA (shown in **(B)**) in the immunoprecipitated materials were determined by quantitative PCR. Results shown are the average of three independent experiments. Asterisks indicate *P* < 0.05 (Mann–Whitney *U* test, SPSS 16.0).

We noted that targeting the *pol* DNA (T5 to T8) also led to significant reduction in GFP expression that is driven by the LTR and viral Tat protein (Figure 3). One possibility is that Cas9 cleavage of the *pol* DNA, as guided by T5, T6, T7 or T8, triggers DNA damage response which causes histone modification (such as H3K9me3) and consequent transcription silencing of adjacent genes [17]. To test this, we treated JLat10.6 cells with control guide RNA or the T5 to T8 HIV-1 guide RNA together with Cas9, followed by ChIP (chromatin immunoprecipitation) analysis of histone modification at HIV-1 LTR and GFP regions. Two types of histone modification were studied, H3K9me3 that marks transcription suppression and H3K36me3 that marks transcription activation [18]. The results of Figure 4 show that the LTR DNA or GFP DNA is enriched by more than 2-fold by the anti-H3K9me3 antibodies in the HIV-1 guide RNA treated cells as compared to the cells that were treated with either the empty vector or gRNA targeting luciferase (T RL). In contrast, neither anti-H3 nor anti-H3K36me3 antibodies enriched HIV-1 LTR DNA or GFP DNA as a result of HIV-1 guide RNA treatment (Figure 4). Interestingly, treatment with the GFP gRNA (T GFP) led to specific enrichment of GFP DNA associated with anti-H3K9me3 antibody; in contrast, HIV-1 LTR DNA was not significantly enriched (Figure 4). These data suggest that, in addition to generate mutations in the target DNA, the CRISPR/Cas9 machinery also induces H3K9me3 histone modification at the target site, which contributes to transcription suppression.

In conclusion, results of this study demonstrate the great potency of the CRISPR/Cas9 system to target and inactivate HIV-1 proviral DNA in the latent JLat10.6 cell line. Since JLat10.6 is a clonal cell line in which HIV-1 is integrated at a specific site in cellular DNA, results in this study cannot conclude whether the CRISRP/Cas9 system may target HIV-1 DNA that is integrated at different cellular DNA loci with different efficiency. Since the CRISPR/Cas9 complex targets both the transcription inert and the transcription active HIV-1 DNA with similar efficiency, CRISPR/Cas9 should be effective in eliminating HIV-1 reservoirs in which HIV-1 exists in a latent state. In support of our data, recent studies reported suppression of the HIV-based vectors using the CRISPR/Cas9 system [19,20]. Both studies tested gRNAs targeting HIV-1 LTR. We have herein tested 10 target sites across HIV-1 genome and identified one site in the second exon of Rev (called T10) that exhibits the highest level of mutation by Cas9. In a separate study, the CRISPR/Cas9 system was exploited to successfully generate the CCR5Δ32 deletion in pluripotent stem cells with 33% efficiency, as compared to 14% efficiency when the TALENS method was employed [21]. Together, all these findings support the utility of the CRISPR/Cas9 system as a new genome editing approach to eradicate HIV-1. Challenges also exist to target a highly mutable virus like HIV-1 using the CRISPR/Cas9 system whose efficacy is largely dependent on how well the gRNA matches the target viral DNA sequence. One solution can be a personalized approach in which the gRNA is designed to match the HIV-1 sequences that are archived in the reservoir of the individual patient. This effort may especially pay off if such an approach can be used to cure infected individuals. A second strategy may involve exploiting multiple gRNAs to target several relatively conserved sites in the HIV-1 genome in order to maximize efficacy and minimize virus escape. With the CRISPR/Cas9 field quickly evolving, new tools will hopefully emerge to help eradicate HIV in clinical settings.
